# Multidirectional Trunk Movements Reveal Hidden Symmetry Loss in Stroke: An Electromyography-Based Comparative Study

**DOI:** 10.3390/medicina61091603

**Published:** 2025-09-05

**Authors:** Hyejin Shin, Taewoong Jeong, Yijung Chung

**Affiliations:** 1 Department of Physical Therapy, Wirye Hospital, Seongnam 13647, Republic of Korea; 2 Department of Rehabilitation Medicine, Soonchunhyang University Bucheon Hospital, Bucheon 14584, Republic of Korea; 3 Department of Physical Therapy, College of Health and Welfare, Sahmyook University, Seoul 01795, Republic of Korea

**Keywords:** stroke, hemiplegia, symmetry, electromyography, trunk movement, trunk control, abdominal muscles, skeletal muscle, postural balance, comparative study

## Abstract

*Background and Objectives*: Stroke and hemiplegia disrupts symmetrical activation of skeletal and abdominal muscles, impairing trunk control and functional movement. Although asymmetry is also present in healthy adults, its magnitude and patterns differ with neurological impairment. Understanding trunk muscle symmetry across functional tasks in healthy individuals and patients with stroke is essential for targeted rehabilitation strategies. *Materials and Methods*: A comparative cross-sectional study was conducted including healthy adults and patients with stroke. Muscle activation symmetry of the rectus abdominis, external oblique, internal oblique, and multifidus was analyzed across four trunk movements: flexion, extension, and lateral flexion to the dominant or non-dominant side. A two-way repeated measures ANOVA examined main and interaction effects of condition, muscle, and group. *Results*: Trunk muscle symmetry was significantly influenced by the movement conditions, and patterns of change differed between groups. While no consistent differences were observed across muscles, specific interactions revealed condition-dependent variations, particularly between abdominal and deep spinal muscles. Lateral flexion elicited the greatest asymmetry, with distinct response patterns in healthy individuals compared with patients with stroke. *Conclusions*: This study highlights the importance of addressing movement-specific demands in trunk rehabilitation. Rather than focusing on isolated muscles, interventions should consider the dynamic and condition-dependent nature of symmetry to optimize functional recovery in patients with stroke.

## 1. Introduction

The trunk muscles are crucial for stabilizing the spine and pelvis and for the active control of the limbs. Control of the trunk muscles supports most daily activities, including sitting, standing, and walking [1]. Specifically, in a sitting position, forward and backward movements are influenced by both trunk and leg muscles, while lateral movements are entirely generated by the trunk muscles [2].

After a stroke, limitations in the trunk and a decrease in muscle strength can occur, leading to difficulties in maintaining body posture, shifting the center of gravity, and movement [3]. Patients with stroke experience asymmetric weight bearing in the sitting position and a reduced ability to shift their center of gravity during forward, backward, or lateral movements [4]. Such impairment in trunk control during sitting positions impacts fall risk, mobility, functional recovery, and patient prognosis [5]. Consequently, to improve functional independence in patients with stroke, it is necessary to enhance trunk control [6].

Previous studies have investigated electromyographic (EMG) activity of the trunk muscles during trunk flexion and extension to identify deficits in neuromuscular control in patients with stroke. Active trunk flexion and extension, being symmetrical movements, are often used to assess differences in neuromuscular coordination between both sides of the body [7]. To compare muscle activity between the paretic and non-paretic sides, a symmetry index has been used. This index reflects the difference in muscle activation between corresponding muscles on both sides [8]. Furthermore, symmetry index values measured during upper and lower limb flexion movements have been reported to correlate with clinical assessments such as the Motor Assessment Scale and Barthel Index [9]

As such, the trunk muscle symmetry index is closely related to clinical outcomes. Previous research has also shown that patients with stroke exhibit lower temporal synchronization between the paretic and non-paretic sides compared to healthy individuals. Temporal synchronization is evaluated using cross-correlation values, which reflect the timing coordination between muscles on both sides or between muscles aligned along the trunk’s axial and lateral directions [10]. Higher cross-correlation values indicate better synchronization of muscle activation timing. While these values provide insight into reduced trunk muscle function in patients with stroke, there remains a lack of research focused on the symmetry of trunk muscle activation during active movement.

Liao et al. [11] compared trunk muscle symmetry between patients with stroke and healthy individuals. They reported that patients with stroke exhibited differences in muscle activity between the paretic and non-paretic sides during trunk flexion and extension and found it easier to maintain balance during flexion than extension. In another study using the Modified Functional Reach Test, researchers observed trunk and upper limb muscle activity in patients with stroke and found muscle contraction on the paretic side even when the non-paretic arm was moved [4]. Particularly, Haas et al. [3] found that patients with stroke showed lower activity in the lateral multifidus, erector spinae, and external oblique muscles during lateral trunk flexion in a sitting position, compared to healthy individuals. However, they also reported no significant difference between the paretic and non-paretic sides of the erector spinae muscle during lateral flexion.

As shown in these studies, the importance of trunk muscles in a sitting position has been well established. While previous studies have analyzed trunk muscle activity using EMG and evaluated symmetry during trunk flexion and extension in static sitting postures, there is a lack of research directly comparing healthy adults and patients with stroke. Research focusing on lateral trunk flexion, in particular, is notably scarce. Therefore, it is necessary to compare trunk muscle activity symmetry during multidirectional trunk movements in a sitting position between healthy adults and patients with stroke.

The purpose of this study is to compare trunk muscle symmetry between healthy individuals and patients with stroke during multidirectional trunk movements in a sitting position. This study could provide foundational data for future trunk control training in stroke rehabilitation.

## 2. Materials and Methods

### 2.1. Participants

The sample size of study participants was determined using G Power 3.1 software (version 3.1.9.7; Heinrich Heine University, Dusseldorf, Germany). Using F-test (ANOVA: Repeated measures, within-between interaction) with an effect size of 0.4, statistical power of 0.95, and significance level of 0.05, the sample size was calculated to be 24. The sample size was determined to be 28, considering a 20% dropout rate.

This study recruited a total of 28 participants, including healthy adults and patients with stroke, through consecutive sampling and was conducted at W Hospital located in Gyeonggi Province. The inclusion criteria for study participants were as follows: For healthy individuals: (1) no orthopedic disorders, (2) no cardiovascular diseases, and (3) no history of trauma or pain. For patients with stroke: (1) diagnosed with first-ever stroke, (2) free from orthopedic disorders that could influence study outcomes, (3) without neurological disorders other than first-ever stroke that could influence study outcomes, (4) capable of maintaining an independent sitting position, and (5) able to follow researchers’ instructions adequately (MMSE-K score of 24 or above). The exclusion criteria were as follows: (1) having undergone orthopedic surgery within the past 6 months, (2) having severe musculoskeletal or cardiovascular diseases, (3) having significant hearing or visual impairments [12].

All participants signed a consent form after the procedure, and the study’s purpose was explained. This research was approved by the Sahmyook University Institutional Review Board (approval number: SYU 2023-05-001-005). The participants had a complete understanding of the study’s aims and methods. The study followed the ethical principles outlined in the Declaration of Helsinki.

### 2.2. Experimental Procedure

This study utilizes a comparative cross-sectional study design. All participants received a thorough explanation of the study’s purpose, methods, and the scope of results usage, and they voluntarily consented to participate in the experiment. Before the experiment, the researcher explained the measurement positions to the participants and ensured that they understood the procedure by having them practice each position at least three times. The experiment was conducted with 28 participants who met the selection criteria. The selected study participants included 14 patients with stroke and 14 healthy adults. The general characteristics of the study participants, including age, height, weight, and dominant side, were investigated in both groups.

This study compared the symmetry of trunk muscle activity during trunk movements in a sitting position. The four different experimental conditions assessing trunk function were trunk flexion, trunk extension, left trunk flexion, and right trunk flexion in a sitting position. Prior to the experiment, the participants drew lots to determine the conditions under which the trunk control muscle activity would be evaluated.

**Figure 1:**
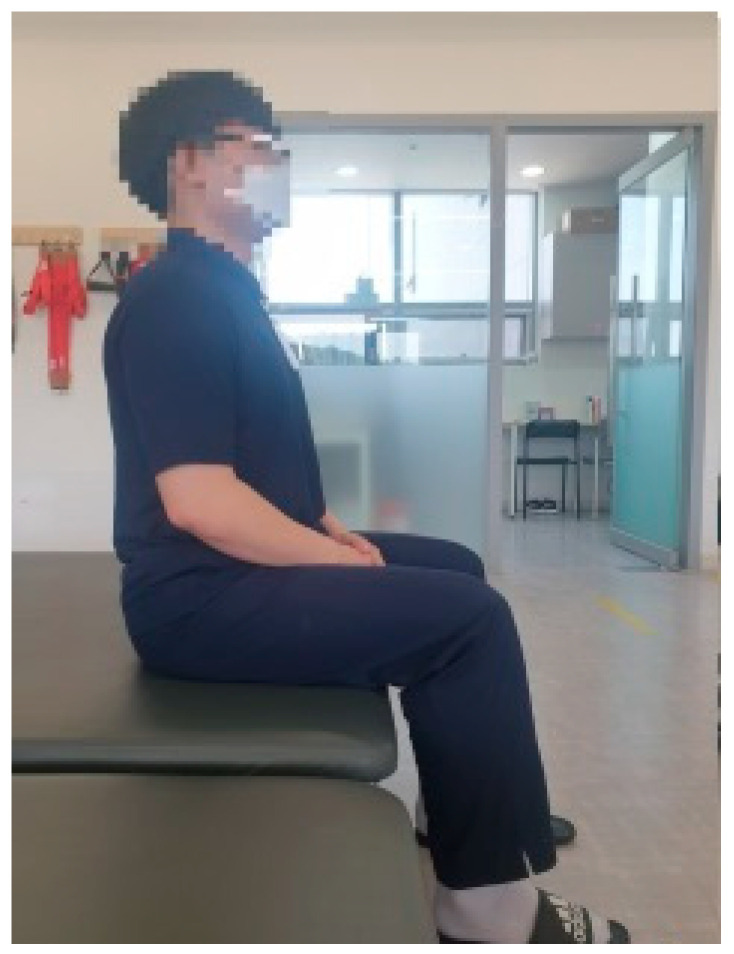
Starting position.

All movements for the experiment were initiated from an upright sitting position, using a chair without a backrest or armrests. In this study, the upright sitting position is defined as sitting with the hips and half of the thighs in contact with the seat, feet flat on the ground, and the back straight. Additionally, with the gaze directed straight ahead, the feet are positioned shoulder-width apart, with the hip and knee joints flexed at 90-degree angles, and the ankle joints in a neutral position (Figure 1). The movements were initiated in response to the therapist’s instructions, and participants were given 2 s to move from the starting position to the end range. Participants were required to hold the position at the end range for 5 s, with no time limit for returning to the starting position. A metronome was used to ensure precise timing. If either the buttocks or feet lifted at the end range, it was considered a failure, and an opportunity for reattempt was given. If the participant experienced three failures, the attempt was recorded as a failure. To prevent muscle fatigue between movements, a 1-min rest period was provided between each movement.

**Figure 2:**
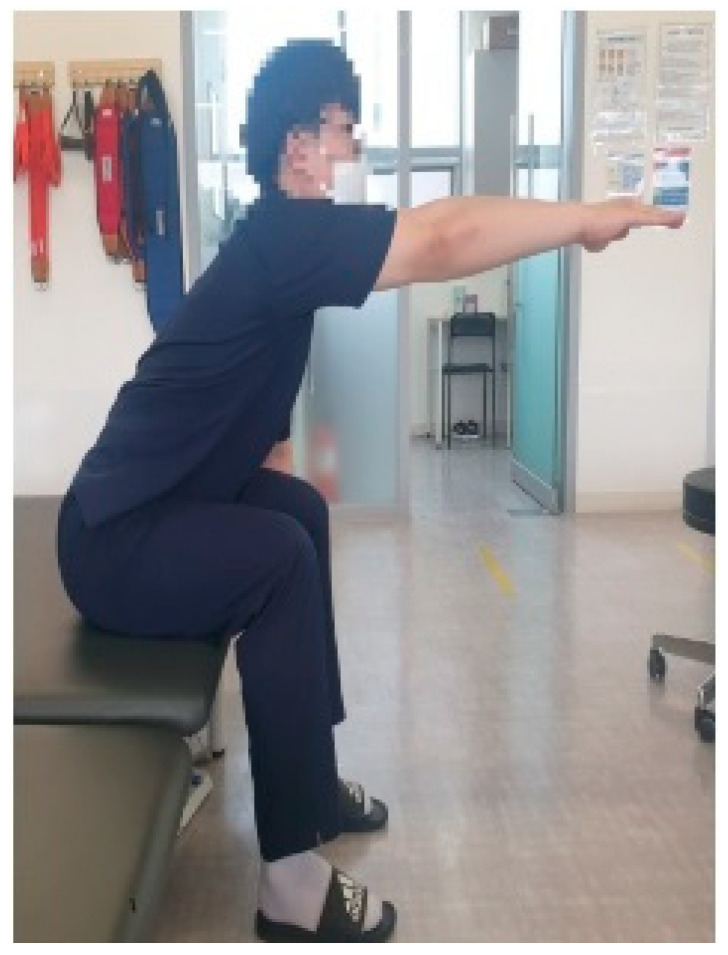
Trunk flexion.

**Figure 3:**
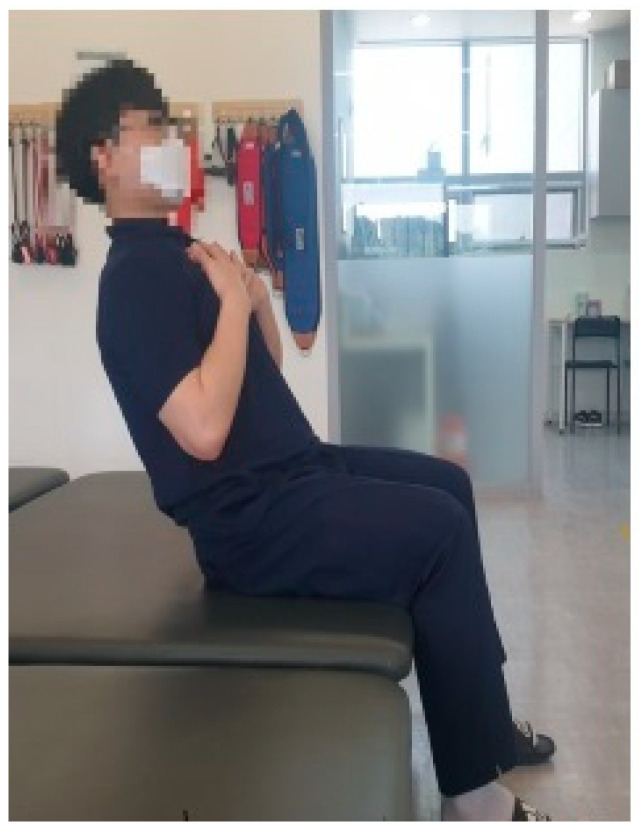
Trunk extension.

**Figure 4:**
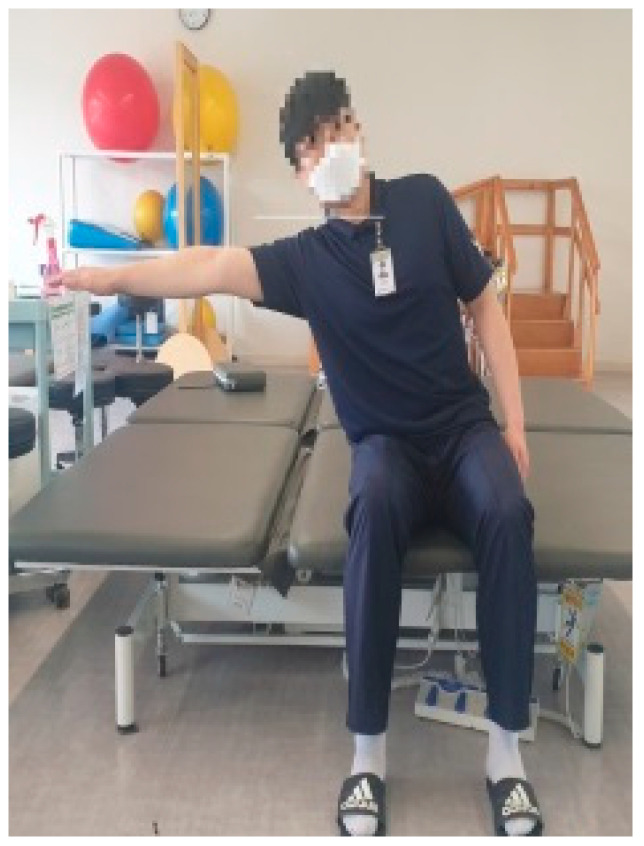
Trunk lateral flexion.

Trunk flexion is defined as extending the dominant arm as far forward as possible while sitting position, without the buttocks lifting off the seat (Figure 2). Trunk extension is defined as leaning the trunk as far backward as possible while sitting position, with both arms gathered at the chest and both feet fixed on the floor (Figure 3). Trunk lateral flexion is defined as extending the same-side arm as far as possible in the direction of lateral flexion while sitting position, with the opposite hip and foot remaining in contact with the seat and floor (Figure 4). In the case of patients with stroke, during lateral flexion of the affected side, the movement was performed with the affected arm placed next to the thigh. The measurements used data from the middle 3 s, excluding the first and last 1 s. Each movement was repeated three times, and the average value was used for analysis.

### 2.3. Outcome Measures

To assess muscle activity, surface electromyography (Noraxon Ultium EMG, Noraxon Inc., Scottsdale, AZ, USA) was utilized. The sampling rate of the electromyography signals was set to 2000 Hz, and the frequency bandwidth was set to 20–500 Hz. In this study, the electromyography signals from the muscles measured were processed using Myoresearch MR3 Master edition software (Noraxon Ultium EMG, Noraxon Inc., Scottsdale, AZ, USA), applying full-wave rectification and obtaining the root mean square (RMS) value over 500 ms. After performing trunk movements for each condition, participants maintained the posture for 5 s. A stable 3 s segment, excluding the initial and final 1 s, was extracted for analysis. At this time, only segments with no visually noticeable noise and that corresponded to the metronome interval were selected. Measurements were repeated three times for each condition, and the mean values were calculated. The symmetry of trunk muscle activation was then determined using the formula (unaffected side − affected side)/(unaffected side + affected side) * 100 [13].

All electrode sites were prepared by shaving, gentle abrasion, and cleansing with alcohol swabs to reduce skin impedance. Electrodes were attached parallel to the muscle fiber direction, maintaining an inter-electrode distance of 20 mm using commercially available bipolar (Ag/AgCl) electrodes. The cables were taped to minimize motion artifacts. Electrode placement was based on previous literature and recommendations [14], and the anatomical landmarks for each muscle followed the locations presented in the manuscript. Attachment was performed in accordance with SENIAM guidelines, standardizing skin preparation, electrode spacing, and placement orientation according to the same protocol. The electrode placement was as follows: For the rectus abdominis, the electrodes were attached 3 cm lateral to the umbilicus. For the external oblique, the electrodes were placed 15 cm lateral to the umbilicus and in the area between the lower ribs and the anterior superior iliac spine. For the internal oblique, the electrodes were attached to the upper middle portion of the inguinal ligament and the anterior superior iliac spine. For the multifidus, the electrodes were placed horizontally above and below the line connecting the posterior superior iliac spines [14].

### 2.4. Data Analysis

The data collected in this study were analyzed using the statistical software PASW Statistics version 18.0 for Windows (SPSS Inc., Chicago, IL, USA). The general characteristics of the participants were summarized using descriptive statistics presented as mean ± standard deviation. The normality of all continuous variables was tested using the Shapiro–Wilk test. A two-way repeated measures ANOVA was conducted to examine the effects of group (patients with stroke vs. healthy individuals) as the between-subjects factor, and movement condition (flexion, extension, lateral flexion) and muscle type as within-subjects factors on trunk muscle symmetry during movement from a sitting position. Post hoc comparisons were performed using the Bonferroni correction to identify specific differences between groups and conditions. The significance level for all analyses was set at 0.05.

## 3. Results

### 3.1. General Characteristics of Participants

The participants in this study consist of 14 healthy adults and 14 patients with stroke. The general characteristics of the participants are shown in Table 1. Healthy adults group consists of 5 males and 9 females, with an average age of 57.78 ± 12.67 years, average height of 165.28 ± 9.98 cm, and average weight of 66.50 ± 13.60 kg. Patient with stroke group consists of 10 males and 4 females, with an average age of 56.42 ± 10.91 years, average height of 167.07 ± 8.31 cm, and average weight of 65.06 ± 11.81 kg.

### 3.2. Comparison of Muscle Activation Symmetry

In this study, we analyzed changes in muscle activation symmetry across four conditions: TF (forward flexion), TE (trunk extension), TN (dominant side lateral flexion for healthy participants, non-paretic side for patients), and TA (non-dominant side lateral flexion for healthy participants, paretic side for patients). Four muscles were examined: RA (Rectus Abdominis), EO (External Oblique), IO (Internal Oblique), and MF (Multifidus). The participants included 14 healthy adults and 14 patients with stroke, and a two-way repeated measures ANOVA was conducted to compare between groups.

Mauchly’s test of sphericity indicated that the assumption of sphericity was violated for condition and condition × muscle; therefore, Greenhouse–Geisser corrections were applied.

A significant main effect of condition was found, F (3, 78) = 40.767, *p* < 0.001, and partial η^2^ = 0.611, indicating that muscle activation symmetry differed significantly across the four movement conditions. The main effect of muscle was not significant, F (3, 78) = 2.134, *p* = 0.103, partial η^2^ = 0.076. However, the interaction between condition and muscle was significant, F (9, 234) = 7.523, *p* < 0.001, partial η^2^ = 0.224. Furthermore, the condition × group interaction was also significant, F (3, 78) = 6.086, *p* = 0.001, partial η^2^ = 0.190, suggesting that the pattern of changes across conditions differed between healthy participants and patients with stroke. In contrast, the muscle × group interaction was not significant, F (3, 78) = 0.841, *p* = 0.476, partial η^2^ = 0.031, nor was the condition × muscle × group interaction, F (9, 234) = 1.435, *p* = 0.212, partial η^2^ = 0.052 (Table 2).

Post hoc analyses with Bonferroni correction were performed to investigate pairwise differences. For condition comparisons, significant differences were observed between TF and TN (mean difference = 19.730, 95% CI [10.445, 29.015], *p* < 0.001) and between TE and TA (mean difference = 33.938, 95% CI [23.355, 44.520], *p* < 0.001). The difference between TF and TE was not significant (mean difference = 4.075, 95% CI [−2.344, 10.495], *p* = 0.203). Among the conditions, the difference between TN and TA was the most pronounced (Table 3).

For muscle comparisons, a significant difference was found between RA and MF (mean difference = 8.135, 95% CI [3.551, 19.821], *p* = 0.042), as well as between EO and IO (Table 4).

In the condition × group interaction post hoc tests, healthy participants showed a significant difference in muscle activation symmetry between TF and TN (mean difference = 10.933, 95% CI [3.032, 18.834]), whereas patients with stroke exhibited a larger difference between TE and TA (mean difference = 20.546, 95% CI [12.645, 28.447]) (Table 5).

## 4. Discussion

In the human body, symmetrical movements are generally considered healthier than asymmetrical ones; however, even healthy adults exhibit a certain degree of asymmetry. It has been reported that lower limb asymmetry is more pronounced during stair climbing compared to walking, and asymmetrical movements are more noticeable during complex tasks than during simple ones [15]. Figas et al. [16] investigated the symmetry of the sternocleidomastoid and upper trapezius muscles during neck movements in healthy adults. They found that these muscles contracted most symmetrically when performing the same movements on both sides of the neck. However, asymmetry in muscle activity was also observed during rest. Yoon et al. [17] measured the bilateral thickness of the transversus abdominis and external oblique muscles in seated adult males using ultrasound. They found a significant difference in muscle thickness between the left and right sides in a static position; however, this difference disappeared when external loading was applied.

Reaching movements accompanied by trunk flexion are commonly used in daily life and can contribute to functional improvement in trunk control. Winzeler-Merçay [18] examined the muscle activity of the rectus abdominis and erector spinae during trunk flexion and extension in patients with stroke. In a study by Kim et al. [19], reaching movements were found to influence not only the entire trunk but also the lower limbs.

In this study, trunk muscle symmetry varied significantly depending on the movement condition, and a condition × group interaction was observed, indicating that the pattern of changes across conditions differed between healthy individuals and patients with stroke. However, during trunk flexion and extension specifically, no group differences were evident, which is consistent with previous findings reporting similar levels of muscle activation symmetry during symmetrical trunk movements in patients with stroke [20]. However, Marchesi et al. [4] measured the activity of trunk muscles and the affected upper limb using a modified Functional Reach Test. They observed symmetrical muscle activity in the external oblique muscles during forward reaching, and muscle activation was also present in the affected upper limb. Additionally, van Criekinge et al. [21] reported that not only trunk muscles but also lower limb muscles are recruited as part of a compensatory mechanism during trunk flexion and extension movements. For these reasons, it is believed that reduced muscle activity in the trunk muscles may have occurred in the present study as well. Ma et al. [22] compared trunk flexion with and without arm extension in healthy individuals and patients with stroke. They found that movements were more pronounced when the arms were extended, and patients with stroke showed significantly greater trunk movement on the affected side.

In the present study, the main effect of muscle was not significant; however, a condition × muscle interaction was found. This suggests that differences in muscle symmetry were not consistent across all muscles but emerged in specific comparisons, such as between the rectus abdominis and multifidus, and between the external and internal oblique muscles. These results partly align with previous studies showing selective differences in trunk muscle activity depending on experience or movement context, such as the greater internal oblique activation reported in individuals with Pilates training [23] or the absence of significant rectus abdominis differences in patients with stroke [24].

This study identified differences in trunk muscle symmetry during trunk movements between healthy individuals and patients with stroke. Previous studies on healthy adults have reported that movements involving lateral flexion or rotation induce asymmetrical muscle contractions, with increased muscle activity observed on the side opposite to the direction of movement. Unlike previous studies in which no significant trunk muscle differences were reported in patients with stroke [16], the present study demonstrated a condition × group interaction. Specifically, healthy individuals showed greater changes in symmetry between forward flexion and lateral flexion, whereas patients with stroke exhibited more pronounced differences between trunk extension and lateral flexion. Although the muscle × group interaction was not significant, these findings indicate that the overall pattern of muscle activation symmetry differs between groups depending on the movement condition. During movements in a seated position, muscle activation tends to be stronger during eccentric contractions. Compared to healthy individuals, patients with stroke showed more symmetrical activation of the internal oblique muscle during movements on the affected side. In a study by Bae [25], muscle activity on the paretic side was reported to be higher during movement, and increased symmetry was observed in the internal oblique during non-dominant side lateral flexion.

In the study by Curuk et al. [26], the muscles on the affected side of patients with stroke showed a tendency for decreased activation, and a significant reduction in symmetry was observed in the external oblique muscle during dominant side lateral flexion. Chen et al. [27] reported that patients with stroke showed decreased consistency in muscle activation during 180-degree rotation and exhibited hyperactivation to compensate for lower limb weakness. They found that the external oblique muscle on the paretic side had higher muscle activation compared to healthy individuals, while the external oblique on the non-paretic side showed no significant difference from healthy controls. In this experiment, during lateral trunk flexion, the external oblique muscle showed higher symmetry when flexing on the paretic side and lower symmetry when flexing on the non-paretic side. The lack of significant differences in trunk muscle symmetry during most movements in patients with stroke is believed to be due to reduced muscle activation caused by brain injury. In particular, the difference between TN and TA was the most pronounced in post hoc analyses, supporting the notion that lateral flexion imposes the greatest asymmetry burden.

This study was a comparative cross-sectional study including healthy individuals and patients with stroke. The research was conducted on patients admitted to a single medical institution, and all patients with stroke who participated were at least at Brunnstrom stage 3. Therefore, the results cannot be generalized to all patients with stroke. Future studies should include patients in the early recovery stages from multiple institutions and investigate the therapeutic effects of trunk control interventions on trunk muscle activation.

This study has several limitations. First, the sample size was relatively small, which may limit the generalizability of the findings. Second, there was an imbalance in gender distribution between groups, which could have influenced muscle activation patterns and symmetry outcomes. Finally, all patients were recruited from a single institution and were in relatively similar stages of stroke recovery, which may not represent the full spectrum of patients. Future studies with larger and more diverse populations are warranted.

## 5. Conclusions

This study emphasizes the importance of addressing movement-specific demands in trunk rehabilitation for patients with stroke. Rather than focusing solely on strengthening individual trunk muscles, it is crucial to consider how muscle activation patterns and symmetry change dynamically depending on the type and direction of movement. The findings revealed that healthy adults demonstrated variable symmetry patterns depending on movement direction, while patients with stroke exhibited reduced and less differentiated muscle activity, particularly during lateral flexion. This suggests that brain injury not only weakens trunk muscles but also impairs the coordinated control required for symmetrical movement. Therefore, it may be beneficial to tailor rehabilitation programs to reflect these functional differences, incorporating multidirectional trunk exercises that promote balanced muscle activation. By recognizing the condition-dependent nature of trunk muscle asymmetry, therapists can design more effective, task-specific interventions that support postural stability, reduce fall risk, and ultimately improve functional independence in patients recovering from stroke.

## Figures and Tables

**Table 1:** General characteristics of subjects (*n* = 28).

Characteristics	Healthy Adults (*n* = 14)	Patients with Stroke (*n* = 14)	χ^2^|t(*p*)
Gender			1.955 (0.061)
Male	5	10	
Female	9	4	
Age (years)	57.78 ± 12.67	56.42 ± 10.91	0.514 (0.612)
Height (cm)	165.28 ± 9.98	167.07 ± 8.31	−0.187 (0.853)
Weight (kg)	66.50 ± 13.60	65.06 ± 11.81	−0.304 (0.764)
Dominant side			
Right	14	4	
Left		10	

Values are mean ± standard deviation. χ^2^: Chi-square test; *p*: *p*-value (statistical significance level).

**Table 2:** Results of two-way repeated measures ANOVA for muscle activation symmetry.

Effect	F(df^1^, df^2^)	*p*	Partial η^2^
Condition	F(3, 78) = 40.767	<0.001	0.611
Muscle	F(3, 78) = 2.134	0.103	0.076
Condition × Muscle	F(9, 234) = 7.523	<0.001	0.224
Condition × Group	F(3, 78) = 6.086	0.001	0.190
Muscle × Group	F(3, 78) = 0.841	0.476	0.031
Condition × Muscle × Group	F(9, 234) = 1.435	0.212	0.052

η^2^ = partial eta squared.

**Table 3:** Post hoc comparisons of muscle activation symmetry across conditions.

Comparison	Mean Difference (95% CI)	*p*	Interpretation
TF vs. TN	19.730 (10.445, 29.015)	<0.001	Significant
TE vs. TA	33.938 (23.355, 44.520)	<0.001	Significant
TF vs. TE	4.075 (−2.344, 10.495)	0.203	Not significant
TN vs. TA	−14.792 (−23.584, −6.000)	<0.001	Largest effect

TF = trunk flexion; TE = trunk extension; TN = lateral flexion (dominant/non-paretic side); TA = lateral flexion (non-dominant/paretic side).

**Table 4:** Post hoc comparisons of muscle activation symmetry across muscles.

Comparison	Mean Difference (95% CI)	*p*
RA vs. MF	8.135 (3.551, 19.821)	0.042
EO vs. IO	5.200 (0.200, 10.200)	0.042

RA = Rectus Abdominis; EO = External Oblique; IO = Internal Oblique; MF = Multifidus.

**Table 5:** Post hoc comparisons for condition × group interaction.

Group	Comparison	Mean Difference (95% CI)	*p*
Healthy	TF vs. TN	10.933 (3.032, 18.834)	<0.05
Stroke	TE vs. TA	20.546 (12.645, 28.447)	<0.001

TF = trunk flexion; TE = trunk extension; TN = lateral flexion (dominant/non-paretic side); TA = lateral flexion (non-dominant/paretic side).

## Data Availability

Data are contained within the article.

## References

[1] Lee M.H., Jang S.H. (2019). The effects of the neck stabilization exercise on the muscle activity of trunk respiratory muscles and maximum voluntary ventilation of chronic stroke patients. J. Back Musculoskelet. Rehabil..

[2] Yoon H.S., Cha Y.J., You J.S.H. (2020). Effects of dynamic core-postural chain stabilization on diaphragm movement, abdominal muscle thickness, and postural control in patients with subacute stroke: A randomized control trial. NeuroRehabilitation.

[3] Haas M.C., Sommer B.B., Karrer S., Jörger M., Graf E.S., Huber M., Baumgartner D., Bansi J., Kool J., Bauer C.M. (2022). Surface electromyographic activity of trunk muscles during trunk control exercises for people after stroke; effect of a mobile and stable seat for rehabilitation. PLoS ONE.

[4] Marchesi G., Ballardini G., Barone L., Giannoni P., Lentino C., De Luca A., Casadio M. (2021). Modified Functional Reach Test: Upper-Body Kinematics and Muscular Activity in Chronic Stroke Survivors. Sensors.

[5] Ahmed U., Karimi H., Amir S., Ahmed A. (2021). Effects of intensive multiplanar trunk training coupled with dual-task exercises on balance, mobility, and fall risk in patients with stroke: A randomized controlled trial. J. Int. Med. Res..

[6] Lee P.Y., Huang J.C., Tseng H.Y., Yang Y.C., Lin S.I. (2020). Effects of Trunk Exercise on Unstable Surfaces in Persons with Stroke: A Randomized Controlled Trial. Int. J. Environ. Res. Public Health.

[7] Dickstein R., Heffes Y., Laufer Y., Ben-Haim Z. (1999). Activation of selected trunk muscles during symmetric functional activities in poststroke hemiparetic and hemiplegic patients. J. Neurol. Neurosurg. Psychiatry.

[8] Dickstein R., Sheffi S., Ben Haim Z., Shabtai E., Markovici E. (2000). Activation of flexor and extensor trunk muscles in hemiparesis. Am. J. Phys. Med. Rehabil..

[9] Dickstein R., Shefi S., Marcovitz E., Villa Y. (2004). Anticipatory postural adjustment in selected trunk muscles in post stroke hemiparetic patients. Arch. Phys. Med. Rehabil..

[10] Dickstein R., Shefi S., Marcovitz E., Villa Y. (2004). Electromyographic activity of voluntarily activated trunk flexor and extensor muscles in post-stroke hemiparetic subjects. Clin. Neurophysiol..

[11] Liao C.F., Liaw L.J., Wang R.Y., Su F.C., Hsu A.T. (2015). Electromyography of symmetrical trunk movements and trunk position sense in chronic stroke patients. J. Phys. Ther. Sci..

[12] Huang M., Pang M.Y.C. (2019). Muscle activity and vibration transmissibility during whole-body vibration in chronic stroke. Scand. J. Med. Sci. Sports.

[13] Sung P.S., O’Sullivan E., Park M.S. (2021). The reaction times and symmetry indices in the bilateral trunk and limb muscles in control subjects and subjects with low back pain that persisted two months or longer. Eur. Spine. J..

[14] Criswell E. (2010). Cram’s Introduction to Surface Electromyography.

[15] Siebers H.L., Alrawashdeh W., Betsch M., Migliorini F., Hildebrand F., Eschweiler J. (2021). Comparison of different symmetry indices for the quantification of dynamic joint angles. BMC Sports Sci. Med. Rehabil..

[16] Figas G., Hadamus A., Błażkiewicz M., Kujawa J. (2023). Symmetry of the Neck Muscles’ Activity in the Electromyography Signal during Basic Motion Patterns. Sensors.

[17] Yoon B., Pyeon H., Kim Y., Hong Y., Lee S. (2018). The relation between abdominal muscle asymmetry and trunk postural stability: An ultrasound imaging study. J. Back. Musculoskelet. Rehabil..

[18] Winzeler-Merçay U., Mudie H. (2002). The nature of the effects of stroke on trunk flexor and extensor muscles during work and at rest. Disabil. Rehabil..

[19] Kim J.H., Lee S.M., Jeon S.H. (2015). Correlations among trunk impairment, functional performance, and muscle activity during forward reaching tasks in patients with chronic stroke. J. Phys. Ther. Sci..

[20] Lee N.G., You J.S.H., Yi C.H., Jeon H.S., Choi B.S., Lee D.R., Park J.M., Lee T.H., Ryu I.T., Yoon H.S. (2018). Best Core Stabilization for Anticipatory Postural Adjustment and Falls in Hemiparetic Stroke. Arch. Phys. Med. Rehabil..

[21] Van Criekinge T., Truijen S., Schröder J., Maebe Z., Blanckaert K., van der Waal C., Vink M., Saeys W. (2019). The effectiveness of trunk training on trunk control, sitting and standing balance and mobility post-stroke: A systematic review and meta-analysis. Clin. Rehabil..

[22] Ma H.I., Lin K.C., Hsieh F.H., Chen C.L., Tang S.F., Wu C.Y. (2017). Kinematic Manifestation of Arm-Trunk Performance during Symmetric Bilateral Reaching After Stroke: Within vs. Beyond Arm’s Length. Am. J. Phys. Med. Rehabil..

[23] Lee K. (2021). The Relationship of Trunk Muscle Activation and Core Stability: A Biomechanical Analysis of Pilates-Based Stabilization Exercise. Int. J. Environ. Res. Public Health.

[24] Pereira L.M., Marcucci F.C., de Oliveira Menacho M., Garanhani M.R., Lavado E.L., Cardoso J.R. (2011). Electromyographic activity of selected trunk muscles in subjects with and without hemiparesis during therapeutic exercise. J. Electromyogr. Kinesiol..

[25] Bae D.Y., Kim S.Y., Park S.R., Oh J.S. (2019). Effects of non-paretic arm movements during bridge exercises on trunk muscle activity in stroke patients. J. Phys. Ther. Sci..

[26] Curuk E., Lee Y., Aruin A.S. (2019). Individuals With Stroke Use Asymmetrical Anticipatory Postural Adjustments When Counteracting External Perturbations. Motor. Control..

[27] Chen I.H., Liang P.J., Chiu V.J., Lee S.C. (2021). Trunk Muscle Activation Patterns During Standing Turns in Patients With Stroke: An Electromyographic Analysis. Front. Neurol..

